# Natural variation in colony inbreeding does not influence susceptibility to a fungal pathogen in a termite

**DOI:** 10.1002/ece3.7233

**Published:** 2021-03-10

**Authors:** Carlos M. Aguero, Pierre‐André Eyer, Jason S. Martin, Mark S. Bulmer, Edward L. Vargo

**Affiliations:** ^1^ Department of Entomology Texas A&M University College Station TX USA; ^2^ Department of Biological Sciences Towson University Towson MD USA

**Keywords:** Blattodea, diversity, *Metarhizium*, *Reticulitermes flavipes*, Rhinotermitidae, social immunity

## Abstract

Reduced genetic diversity through inbreeding can negatively affect pathogen resistance. This relationship becomes more complicated in social species, such as social insects, since the chance of disease transmission increases with the frequency of interactions among individuals. However, social insects may benefit from social immunity, whereby individual physiological defenses may be bolstered by collective‐level immune responses, such as grooming or sharing of antimicrobial substance through trophallaxis. We set out to determine whether differences in genetic diversity between colonies of the subterranean termite, *Reticulitermes flavipes*, accounts for colony survival against pathogens. We sampled colonies throughout the United States (Texas, North Carolina, Maryland, and Massachusetts) and determined the level of inbreeding of each colony. To assess whether genetically diverse colonies were better able to survive exposure to diverse pathogens, we challenged groups of termite workers with two strains of a pathogenic fungus, one *local* strain present in the soil surrounding sampled colonies and another *naïve* strain, collected outside the range of this species. We found natural variation in the level of inbreeding between colonies, but this variation did not explain differences in susceptibility to either pathogen. Although the naïve strain was found to be more hazardous than the local strain, colony resistance was correlated between two strains, meaning that colonies had either relatively high or low susceptibility to both strains regardless of their inbreeding coefficient. Overall, our findings may reflect differential virulence between the strains, immune priming of the colonies via prior exposure to the local strain, or a coevolved resistance toward this strain. They also suggest that colony survival may rely more upon additional factors, such as different behavioral response thresholds or the influence of a specific genetic background, rather than the overall genetic diversity of the colony.

## Graphical abstract

Reduced genetic diversity through inbreeding can negatively affect pathogen resistance. We investigated this hypothesis in termites by testing whether the level of inbreeding within a colony affected the survival of workers challenged with two fungal pathogens. We found a bimodal distribution of colony survival, whereby colonies exhibit either high or low susceptibility. However, this difference was not explained by the level of inbreeding within the colony, suggesting that colony immunity may rely more on a specific genetic background, rather than its overall genetic diversity.

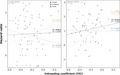

## 1 INTRODUCTION

The loss of genetic diversity through inbreeding can have strong negative effects on the fitness of an organism. Inbreeding depression may result in a reduced immune response to pathogens, which has been a prominent area of concern in populations with exceptionally low genetic diversity, such as in agricultural monocultures (Zhu et al., 2000) and endangered species (O'Brien et al., 1985). The effects of inbreeding on disease dynamics in social species, however, may be more complex. On the one hand, social species may be more prone to disease outbreaks as group living increases the frequency of interactions between host organisms, and therefore, the risk of disease transmission. On the other hand, these species may benefit from herd immunity, whereby enough resistant individuals in a group may reduce the transmission of a disease (Anderson and May 1985). In most eusocial species, all members of a group (*i.e*., colony) arise from the reproduction of a small number of breeders, sometimes only a single queen mated to a single male. Thus, in addition to living in densely packed groups, these species must cope with high relatedness among group members, which may hamper herd immunity if related individuals are more likely to succumb to the same disease.

To date, most social immunity studies have focused on social insects, and more specifically on eusocial Hymenoptera (*i.e*., social bees, wasps, and ants). In this group, colony resistance to pathogens is often associated with intracolony genetic diversity, as genetically distinct individuals may vary in their susceptibility to different disease strains (van Baalen & Beekman, 2006; Bourgeois et al., 2012; Denier & Bulmer, 2015; Evison et al., 2013; Lee et al., 2013; Palmer & Oldroyd, 2003; Shykoff & Schmid‐Hempel, 1991). Genetic diversity within colonies disrupts genotype × genotype interactions (*i.e*., restores the benefits from herd immunity), as a pathogen that can infect one host genotype, may be unable to spread if its next host is resistant. Also, genetically distinct individuals may differ in their propensities to detect, survive, and respond to different pathogens, theoretically making genetically diverse colonies better protected against a diverse array of disease agents (van Baalen & Beekman, 2006; Hamilton, 1987; Schmid‐Hempel, 1998; Sherman et al., 1988). Inversely, increased genetic diversity within a colony may also facilitate infections from a broader range of pathogens (Anderson and May 1985, van Baalen & Beekman, 2006). However, as only a fraction of a genetically diverse colony will be susceptible to a single pathogen genotype, the cost per infection is reduced and may not reach the point of endangering the overall survival of the colony (van Baalen & Beekman, 2006). In social Hymenoptera, several species are able to increase genetic diversity within a colony by increasing the number of breeders. Empirical evidence in honeybees (Bourgeois et al., 2012; Evison et al., 2013; Lee et al., 2013; Mattila et al., 2012; Palmer & Oldroyd, 2003; Seeley & Tarpy, 2006; Tarpy, 2003; Tarpy & Seeley, 2006), bumblebees (Baer & Schmid‐Hempel, 1999; Baer & Schmid‐Hempel, 2001; Liersch & Schmid‐Hempel, 1998), and ants (Hughes & Boomsma, 2004; Reber et al., 2008) shows that increased genetic diversity does indeed improve pathogen resistance at the colony level.

In termites, the relationship between genetic diversity and immunity is less clear. Unlike many Hymenoptera, termites generally do not exhibit variation in their initial number of breeders within a colony. Instead, colonies are typically founded by a single pair of reproductives—a single primary king and queen (simple family)—although multiple primary reproductives have been reported in a growing number of termite species (Hacker et al., 2005; Hartke & Rosengaus, 2013; Montagu et al., 2020; Thorne, 1984). The level of inbreeding within a colony founded by a monogamous pair of reproductives is initially determined by the relatedness between the founders (Eggleton, 2010; Nutting, 1969). Yet, genetic diversity within those colonies may be altered afterward by changes in the colony breeding system (Vargo, 2019). Many termite species may exhibit extended family colonies, where secondary reproductives (*i.e*., neotenic) develop from the colony's offspring when one or both of the founding primary reproductives dies (Myles, 1999). Although secondary reproductives reach sexual maturity, they never develop functional wings and do not leave the colony. Therefore, the reproduction of neotenics extends the life of a colony that would otherwise collapse, at the expense of the colony becoming more inbred over time. Mixed family colonies occur when two separate termite colonies fuse together (Adams et al., 2007; Aguero et al., 2020; Deheer & Kamble, 2008; DeHeer & Vargo, 2004; Fisher et al., 2004; Korb & Schneider, 2007; Perdereau et al., 2010; Thorne et al., 2003). Genetic diversity usually increases in mixed families, depending on the relatedness of the two original colonies. Potentially, the reproductives of both colonies can also interbreed and therefore create new genotypic combinations in the worker force (DeHeer & Vargo, 2008; Johns et al., 2009) . Thus, in most lower termite species, genetic diversity within colonies is initially limited by having only two founders, but diversity can either decrease (in extended families) or increase (in mixed families) over time. However, the degree to which genetic diversity within a colony affects immunity in termites has still not been thoroughly investigated. In the subterranean termite *Reticulitermes flavipes* and the dampwood termites of the genus *Zootermopsis*, colonies can vary dramatically in their susceptibility to different pathogens (Denier & Bulmer, 2015; Rosengaus et al., 2003). However, social immunity of *R. flavipes* is not improved when genetic diversity is artificially increased by creating mixed families in the laboratory (Aguero et al., 2020). Inbreeding was suggested as one of the numerous factors driving incipient colonies of *R. flavipes* to collapse, as the proportion of sibling‐founded mature colonies is significantly lower that the proportion of siblings pairing after a mating swarm, suggesting that inbred colonies did not survive over time (DeHeer & Vargo, 2006). In *Z. angusticollis*, colonies founded by sibling reproductives carried higher microbial loads on their cuticle compared to outbred colonies, presumably due to reduced grooming or a less diverse range of antimicrobials (Calleri et al., 2006). Yet, despite indirect evidence suggesting a lower survival of inbred colonies, the factors driving inbred colonies to collapse and the mechanisms underlying improved pathogen resistance through increased within‐colony genetic diversity remain unclear.

The difficulty in determining the mechanisms influencing pathogen resistance in these species may stem from their complex “social immunity,” whereby overall colony survival is influenced by physiological and behavioral factors at both individual and collective levels (Cremer et al., 2007, 2018; Liu et al., 2019; Traniello et al., 2002). Individual‐level defenses of social insects are the same as those exhibited by solitary insects, like cellular encapsulation (Calleri et al., 2009; Chouvenc et al., 2009a, 2009b; Chouvenc et al., 2011), phagocytosis, and phenoloxidase activity (Rosengaus & Reichheld, 2016). Individual‐level defenses also include the production of defensive compounds (Brown, 1968; Bulmer & Crozier, 2004, 2006; Hölldobler & Engel‐Siegel, 1984; Ortius‐Lechner et al., 2000; Rosengaus et al., 2013; Turillazzi et al., 2006) and pathogen avoidance (Epsky & Capinera, 1988; Marikovsky, 1962; Yanagawa et al., 2012). In addition, social insects display collective immune responses based on interactions between at least two individuals, such as allogrooming (Chouvenc et al. 2009a, Drees et al., 1992; Hughes et al., 2002; Liu, Wang, et al., 2019; Oi & Pereira, 1993; Peng et al., 1987; Rosengaus et al., 1998; Wilson‐Rich et al., 2007; Yanagawa & Shimizu, 2007), the transfer of antimicrobial substances through trophallaxis (Hamilton et al., 2011), their deposition on nest chambers and galleries (Aguero et al., 2021; Chouvenc et al. 2013; Rosengaus et al., 1998), and nest hygiene (Ballari et al., 2007; Bot et al., 2001; Hart & Ratnieks, 2002; Howard & Tschinkel, 1976; Julian & Cahan, 1999; Siebeneicher et al., 1992; Sun & Zhou, 2013; Trumbo et al., 1997). Thus, immunity of social insects relies on complex interactions between individual and collective‐level responses.

In this study, we aimed to determine whether natural levels of genetic diversity affect the susceptibility of *R. flavipes* to pathogens. We sampled termite workers from mature colonies across eight sites distributed throughout four states in the eastern US, where this species exhibits variation in the proportion of family types and the level of within‐colony inbreeding found within populations (Bulmer et al., 2001; DeHeer & Kamble, 2008; Jenkins et al., 1999; Majid et al., 2018; Vargo et al., 2013). To assess whether genetically diverse colonies had better survival toward diverse pathogens, we challenged groups of workers from each colony with two strains of a fungal pathogen, one “local” strain present in the soil surrounding sampled colonies and another “naïve” strain, collected outside the range of this species. We used molecular markers to determine the family type and level of inbreeding within each colony and determined their influence upon colony survival.

## 2 METHODS

### 2.1 Termite sampling

**Figure 1:**
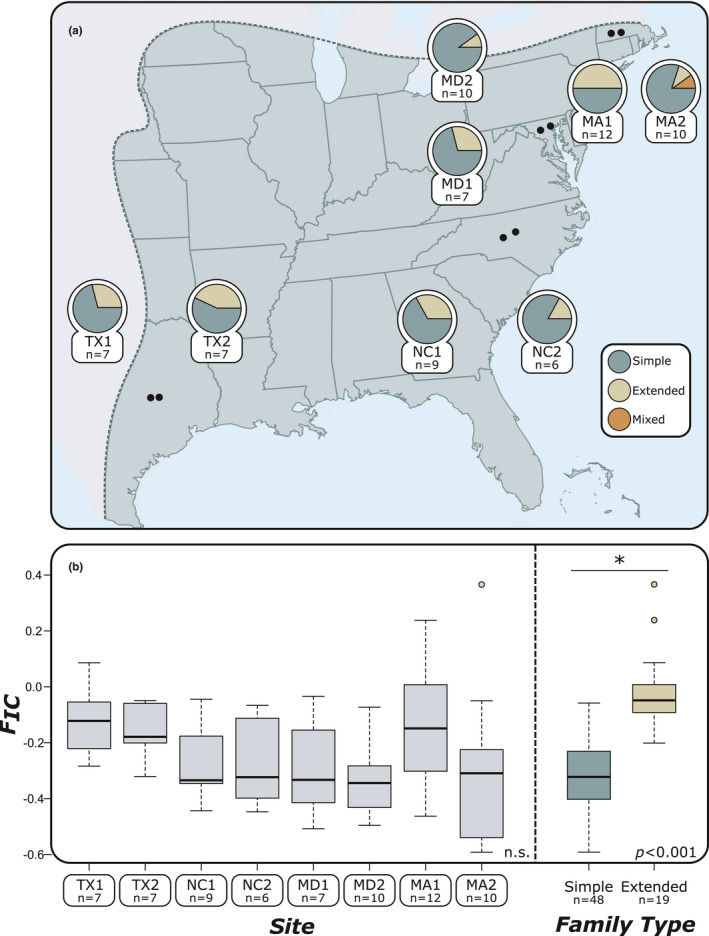
(a) Location of termite sampling sites within the native distribution of *Reticulitermes flavipes* (shaded area of map). Pie charts indicate the proportion of different family types identified from the colonies (*n* = 69 colonies) collected from each site. (b) There was no significant difference found in colony levels of inbreeding (*F*_IC_) between sites (*n* = 69 colonies, *p* = .536). Extended families had significantly higher *F*_IC_ than simple families (*n* = 69 colonies, *p* < .001). Mixed families were not included in the analysis as only one was found

Groups of termite workers were collected from 69 colonies spread among eight sites from Texas (TX1 & TX2), North Carolina (NC1 & NC2), Maryland (MD1 & MD2), and Massachusetts (MA1 & MA2). All collections were made during the summer of 2015. From each colony, 72 workers were kept alive for pathogen bioassays and 20 were directly stored in 100% ethanol for subsequent genetic analyses. The location of sites and the number of nests collected in each site are summarized in Figure 1 and Supplementary Table T1. Within each site, all nests were separated from each other by at least 15 m, as this distance is sufficient to ensure that each nest represents a distinct colony (DeHeer et al., 2005; DeHeer & Vargo, 2004; Vargo, 2003).

### 2.2 Pathogen sampling and bioassay

The immune response of each colony was determined by testing groups of termite workers against two entomopathogenic fungal strains and a control solution. Both pathogens used were strains of *Metarhizium brunneum* collected in this study. Soil samples were collected, and pathogens were isolated from soil using a mealworm baiting assay (Denier & Bulmer, 2015; Hughes et al., 2004). Fungal isolates were identified following the molecular methodology of Denier and Bulmer (2015). One of these strains was isolated from soil collected at a site from which termites were sampled (site MD1) and is referred to in this study as the “local” strain. The other strain was isolated from soil collected in Huntly, Virginia at elevations where *R. flavipes* was not found and is referred to as “naïve” in our study. Both pathogen strains were prepared at the concentration of 1 × 10^7^ conidia/ml in a 0.1% TWEEN®80 (Sigma‐Aldrich Chemie N.V, The Netherlands) conidia suspension. The control treatment was the 0.1% TWEEN®80 solution by itself. From each colony, two replicate groups of 12 termite workers (*n* = 24 workers) were exposed to each treatment solution. Each group of 12 workers was placed in 60 mm petri dishes that were lined with filter paper (Whatman Grade 5, porosity 2.5 μm) moistened with 300 μl dH_2_O for two days prior to the start of the experiment. After this acclimation period, the filter paper was replaced with a new filter paper treated with 300 μL of either the local, the naïve, or the control solution. After 24 hr of exposure, the treated filter paper was replaced with filter paper that had been moistened with 300 μL dH_2_O and termite survival was monitored for 20 days.

### 2.3 Genetic analyses

For each colony, family type and level of inbreeding were determined using DNA from 20 termite workers extracted by a modified PureGene extraction protocol (Supplementary Information S1). Extracted DNA was amplified at nine microsatellite loci that have been previously developed for this species (Dronnet et al., 2004; Vargo, 2000). Microsatellite markers, PCR conditions, and multiplex arrangements are described in Supplementary Information S1. Amplicons were visualized on an ABI 3500 capillary sequencer against a LIZ500 internal standard (Applied Biosystems) and scored using the software Geneious v.9.1 (Kearse et al., 2012). The inbreeding coefficient *F*_IC_ and observed heterozygosity of each colony were calculated using the software packages FSTAT (Goudet, 1995) and GENEPOP (Raymond, 1995). *F*_IC_ estimates the homozygosity of individuals within a social insect colony and is analogous to *F*_IS_ (Bulmer et al., 2001; Thorne et al., 1999; Vargo, 2003). To account for genetic differences between sites, *F*_IC_ was calculated separately for each site. The family type of each colony was determined by observing the number and frequency of alleles within each colony. Colonies with more than four alleles at a locus were classified as mixed families, as more than two unrelated reproductives would be necessary to produce this result. Colonies that had no more than four alleles at a locus but had genotypic combinations that were inconsistent with a monogamous pair of reproductives (for example, an allele paired with itself and two others) were classified as an extended family. When colonies had no more than four alleles at a locus and genotypic combinations typical of a simple family, a G‐test was used to determine whether the frequency of genotypes observed was significantly different from what would be expected from a simple family (Vargo, 2003). Colonies that differed significantly were categorized as extended families, and those that did not were labeled as simple families.

### 2.4 Statistical analyses

Hazard ratios of both pathogen strains were calculated for each colony using a Cox proportional‐hazards model. For each strain, hazard ratios were obtained by comparing the survival of workers between the different colonies. Therefore, these hazard ratio values denote the relative susceptibility of a given colony relative to other colonies. For each pathogen strain, a linear regression was performed to determine the relationship between *F*_IC_ within a given colony and its hazard ratio. Similarly, a linear regression was performed for each strain to assess the relationship between observed heterozygosity within a colony and its hazard ratio. A generalized linear model was used to determine whether *F*_IC_ and family type individually influenced the hazard ratios or if there were any interaction effects. The effects of site and family type on *F*_IC_ were also determined with an analysis of variance (ANOVA), separately. We also compared the hazard ratios of both pathogen strains with a linear regression to determine whether colonies were consistent in their susceptibility to both pathogen strains. To test for difference in colony survival between the two strains, as well as between the control and each strain, termite mortality was analyzed using a log‐rank test under a Cox proportional hazard model. Analyses were performed in the statistical software R 3.5.0 (R Core Team, 2018) using the *survival* package (Therneau & Lumley, 2015).

## 3 RESULTS

Simple and extended families were found in every site, but a majority of the collected colonies were simple families. The proportion of simple families found within each site ranged from 57.14% (site TX2) to 85.71% (site TX1) (Figure 1a). Only a single mixed family was found (site MA2). Overall, the level of inbreeding within each colony (*F*_IC_) ranged between −0.599 and 0.262 (both from site MA2). There was no significant difference in F_IC_ between sites (*p* = .536; Figure 1b). As expected, *F*_IC_ did differ between simple families (*F*_IC_ ± *SD* = −0.326 ± 0.121) and extended families (*F*_IC_ ± *SD* = −0.075 ± 0.157) (*p* < .001; Figure 1b).

**Figure 2:**
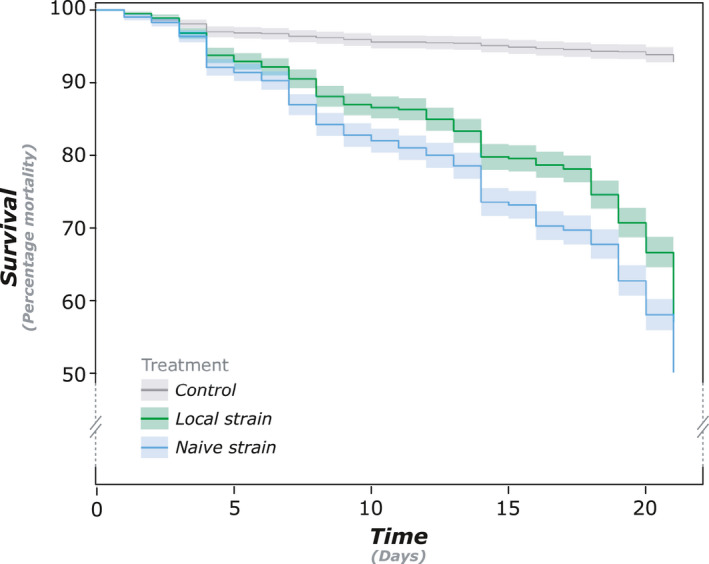
Kaplan–Meier survival distributions of colony groups exposed to a control solution, or a conidia suspension containing either a local or a naïve pathogen strain. Termites exposed to both pathogen strains had significantly lower survival than termites exposed to a control solution (both *p* < .001). Termites exposed to the naïve pathogen strain had significantly lower survival than termites exposed to the local strain (*p* < .001)

**Figure 3:**
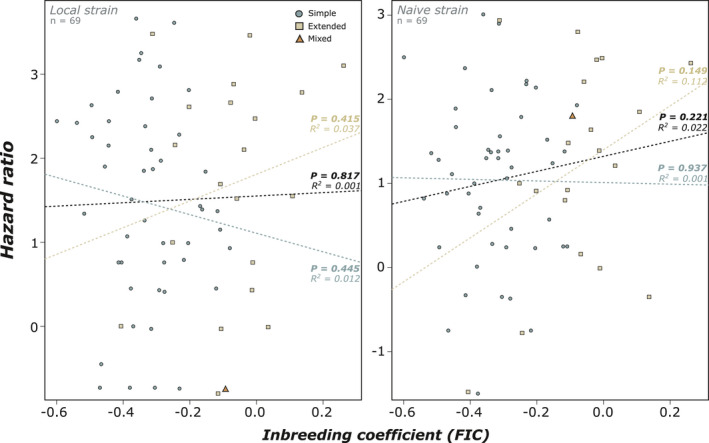
No significant correlation was found between the level of colony inbreeding (*F*_IC_) and the hazard ratio for the local strain (*n* = 69 colonies, *p* = .817) or the naïve strain (*n* = 69 colonies, *p* = .221). There was no significant correlation between the local (*p* = .445 and *p* = .415) and the native strain (*p* = .937 and *p* = .149) when the colonies were separated into simple (*n* = 48 colonies) and extended families (*n* = 19 colonies), respectively

**Figure 4:**
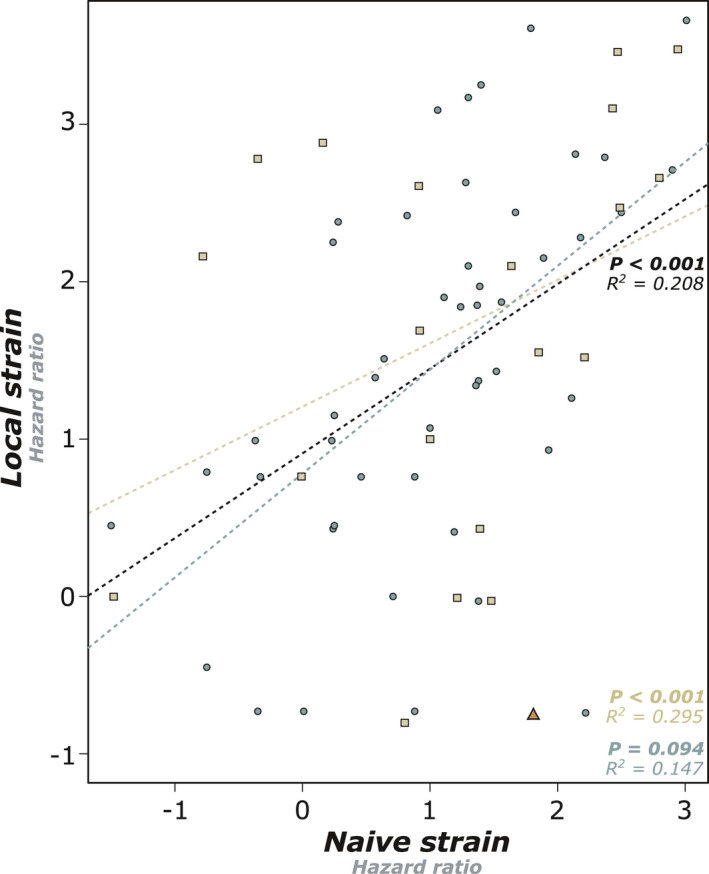
Workers from different colonies were consistent in their hazard ratios for both pathogen strains, such that workers from a colony that was susceptible to the local strain were also susceptible to the naïve strain (*n* = 69 colonies, *p* < .001)

We found a clear effect of both pathogen treatments on colony survival as the local and naive strains respectively kill 60% and 50% of the individuals within groups after 20 days (both *p* < .001; Figure 2). In contrast, 95% of individuals were still alive after 20 days when treated with the control solution (Figure 2). No significant correlation was found between *F*_IC_ and the susceptibility of colonies to either pathogen (Local: *p* = .817; Naïve: *p* = .221; Figure 3a). No significant correlation between *F*_IC_ and the susceptibility of colonies was found when colonies were separated into simple families (Local: *p* = .445; Naïve: *p* = .937) and extended families (Local: *p* = .415; Naïve: *p* = .149; Figure 3). However, despite being nonsignificant, a trend of increasing mortality in inbred extended families was observed for both strains. There were no significant correlations between observed heterozygosity and either pathogen, whether colonies were separated by family type or not (Supplementary Information S2). Similarly, the generalized linear model showed an absence of significant individual or interaction effects from family type and level of inbreeding for both strains (Supplementary Table S2). Although there were no clear main effects of any one variable we recorded, we do report that the naïve strain of *Metarhizium* was significantly more lethal (25%) than the local strain at the same concentration (*p* < .001; Figure 2). Interestingly, although colonies show variable level of susceptibility to both strains of pathogens (Figure 3), we found that colony hazard ratios to both pathogens correlated with each other, such that a colony that was susceptible to one pathogen was also susceptible to the other, and *vice versa* (*p* < .001; Figure 4).

## 4 DISCUSSION

There is natural variation in the level of inbreeding among colonies of *R. flavipes* and is higher in extended families. However, the level of inbreeding did not influence the susceptibility of workers to two strains of a pathogenic fungus. Similarly, pathogen resistance to these strains was not influenced by family type. However, workers from different colonies showed a variable level of susceptibility, which is consistent across the two strains of pathogens tested. These findings suggest that additional factors influence colony survival, such as immune priming through previous exposure, or due to genetic background influencing specific immune behaviors or individual‐level defenses pathways (Chouvenc et al., 2011; Cole et al., 2020; Denier & Bulmer, 2015; Hamilton et al., 2011; Rosengaus, Maxmen, et al., 1998; Rosengaus et al., 1999; Traniello et al., 2002).

### 4.1 Overall genetic diversity does not influence colony survival

Termite colonies are highly variable in their susceptibility to pathogens, such that a pathogen strain that negatively affects one colony may be harmless against another colony (Denier & Bulmer, 2015; Rosengaus, Maxmen, et al., 1998). In *R. flavipes*, the difference in virulence among colonies correlates with the genetic distance between strains of *Metarhizium*, suggesting that genetically similar pathogens have similar virulence depending on the workers' colony of origin (Denier & Bulmer, 2015). This variation in susceptibility may be explained by genotype x genotype interactions, where some colonies consist of more resistant genotypes toward specific pathogen strains or genera. This may account for the variation in colony survival, regardless of their level of inbreeding. This finding suggests that pathogen resistance may rely on specific genetic combinations rather than solely on genetic diversity, as workers from low susceptibility colonies may have 100% of the most resistant genotype, despite being highly inbred (van Baalen & Beekman, 2006). If pathogen resistance is genetically based, the clear separation between high and low susceptibility observed in our study suggests that resistance is influenced only by specific loci. Additionally, alleles influencing pathogen susceptibility may be dominant, as recessive alleles would have likely resulted in a correlation between pathogen susceptibility and inbreeding, leading to the expression of recessive alleles. Potentially, the loci involved in pathogen resistance may code for specific immune behaviors (social immunity), for the production of defensive compounds or cellular encapsulation pathways (individual immunity). Genomic studies have shown that termites carry a full repertoire of immune genes, including all immune‐related pathways present in *Drosophila melanogaster*, such as pattern recognition, signaling, and gene regulation (Korb et al., 2015; Terrapon et al., 2014).

### 4.2 Potential influence of molecular and physiological immune mechanisms

Individual physiological responses of termites include the production of gram‐negative binding proteins (GNBPs), some of them being termite‐specific and different antimicrobial peptides (AMPs), such as attacin, diptericin, termicins, and β‐1,3‐glucanases (Bulmer et al., 2009; Da Silva et al., 2003; Lamberty et al., 2001). Individual‐level defenses also include phagocytosis, the phenoloxidase cascade, and cellular encapsulation. In our study, variation in survival among colonies may illustrate differences in the level of cellular encapsulation (Calleri et al., 2009; Chouvenc et al., 2009b), difference in phenoloxidase activity (Rosengaus & Reichheld, 2016), or difference in the quantity of defensive compounds produced between the different colonies. It may also be explained by differences in the type of defensive compound produced, and their differential effectiveness toward specific strains or genera of pathogens. In our study, we used two strains of the same genus, *Metarhizium*, and found a strong correlation between the hazard ratios of the two strains, meaning that workers from colonies resistant to the local strain also had resistance against the naïve strain. Therefore, colonies with individuals producing a high dose of an effective defensive compound toward this fungal genus would have greater resistance than those with individuals producing a high dose of an ineffective compound or a broad variety of compounds. Interestingly, the genes coding for antimicrobial peptides (*i.e*., termicin) show unusually strong signatures of adaptive evolution in *Reticulitermes* and *Nasutitermes* species, suggesting that the shift to a subterranean lifestyle may have intensified positive selection on these genes (Bulmer & Crozier, 2004, 2006; Bulmer et al., 2010). Overall, this suggests that GNBPs and termicin may play complementary effector roles that could target different fungal pathogens. Different species, especially those with different nesting and foraging habitats, may face distinct selective pressures from different fungal pathogens and have therefore evolved distinct antifungal strategies (Korb et al., 2015; Terrapon et al., 2014).

### 4.3 Potential influence of immune priming

Several termite species may be able to prime their immune defenses against pathogens Rosengaus, Traniello, et al., 1999; Rosengaus et al., 2007). Notably, immune priming does not function in the same way as an adaptive immune system, which acts as an immune memory protecting the organism from subsequent exposures to the same pathogen (Janeway et al., 1999). Immune priming prepares the insect's immune system to be more responsive against any imminent pathogenic threat. Activating innate immune responses can carry high fitness costs, so it is crucial that immune priming only occurs when the host organism is under threat (Schmid‐Hempel, 2005). In our study, the immune priming of some colonies (*i.e*., more resistant) shortly before we performed pathogen assays may account for the variation in colony survival. The previous exposure of these colonies before collection may have allowed them to anticipate future pathogenic threats, such as the ones we applied in our experiments. In termites, the efficiency of immune priming relies on how well individuals can detect nearby pathogens. In *Z. angusticollis*, individuals that have been previously challenged with nonliving pathogenic bacterial cells show improved resistance to live pathogen treatments, which lasts for several days (Rosengaus, Traniello, et al., 1999). Immune priming can also be triggered in individuals that have never been exposed to the pathogen and have only come into contact with pathogen‐challenged nestmates (Traniello et al., 2002). Additionally, *Z. angusticollis* offspring show increased transcription of immune genes when parents have been previously challenged with a pathogen, indicating that termites may be engaging in transgenerational immune priming (Cole et al., 2020). In *R. flavipes*, the presence of pathogen components within nests’ gallery walls after their degradation may prime nearby termites against subsequent infection attempts (Hamilton, Lay, et al., 2011). In addition, variation in colony survival may be explained by differences in detection abilities. The apparent failure of groups of *R. flavipes* workers to quickly detect specific *Metarhizium* strains results in higher mortality (Bulmer et al., 2019). At the collective‐level, pathogen detection alerts nestmates to begin social immune responses, such as allogrooming or cannibalism, whereas at the individual‐level, detecting nearby pathogens may initiate immune priming. Collective‐level defenses may even alleviate some of the costs of individual immunity, as immune gene expression in *R. chinensis* is lower when workers are challenged as a group so they can groom each other (Liu, Wang, et al., 2019). In this study, although we do not have any measure of the termites’ ability to detect pathogens, the increased mortality toward the naïve strain could potentially reflect an inability to detect and to prime their immunity to that specific strain, rather than the shortcomings of defensive compounds. Interestingly, the decreased survival toward the local strain can also represent a coevolved resistance (regardless of the genetic diversity) while colonies exposed to the naïve strain having had no opportunity to evolve strain‐specific resistance. However, the correlation between the hazard ratios of the two strains may rule out this possibility, as such correlation is not expected if colonies exhibit coevolved resistance toward one strain but not toward the other. The difference in colony survival against the two strains may also simply denote a difference of virulence between the two strains, with some colonies being better equipped to detect or defend themselves against *Metarhizium* as a whole.

### 4.4 Potential influence of behavioral defense

Beyond immune priming, pathogen detection ability also influences behavioral defenses. When termites detect pathogens, they avoid infected areas (Epsky & Capinera, 1988; Yanagawa et al., 2012, 2015). Further, individual termites can alert nestmates to nearby pathogens through vibratory alarms (Rosengaus et al., 1999). The experiment in this study was designed so that termites must walk on a substrate that has been treated with pathogenic conidia. By detecting and communicating the presence of nearby conidia, workers could alert their nestmates to reduce their own movement on the substrate and effectively reduce the number of conidia to which they are exposed. In addition, while individual termites can groom themselves, conidia that accumulate in difficult‐to‐reach parts of the body are most effectively removed through allogrooming (Rosengaus, Maxmen, et al., 1998, Shimizu & Yamaji, 2003, Yanagawa & Shimizu, 2007, Yanagawa et al., 2008, Chouvenc et al. 2009a, Davis et al., 2018, Bulmer et al., 2019). Therefore, the difference in colony survival may be explained by different colonies of origin exhibiting variable levels of pathogen detection, grooming behavior, and avoidance. However, in comparison with field conditions, this experimental setup also evolves small group of termites, which may disturb these aspects of social immunity emerging more properly in large group within their nest/foraging environment. Similarly, the initial microbial loads of the colonies tested were not measured. These potentially variable loads of microbes present before the experiment may directly or indirectly interact with the pathogens tested and thus hamper the assessment of survival of the colonies toward the focal pathogen.

### 4.5 Potential influence of colony age

Interestingly, a nonsignificant trend was observed in extended families between colony inbreeding and the hazard ratio of both pathogen strains. Termite colonies do not become extended families until one or both of the primary reproductives in the colony dies (Myles, 1999). Thus, the transition to an extended family is more likely to occur in older colonies. Additionally, the level of inbreeding within extended families increases over time with additional turnovers of neotenic reproductives. The correlation between inbreeding and survival in extended colonies may therefore suggest that inbreeding depression, or additional factors, only becomes relevant as a colony ages. Similarly, the lack of correlation between inbreeding and colony survival in simple families suggests that the reduced foundation survival in inbred colonies observed in the field (Deheer & Vargo, 2006) is not caused by a putative reduced immunity. Similarly, our findings using mature colonies contrast with previous results on incipient colonies of the dampwood termite *Zootermopsis angusticollis,* where sibling reproductive pairs had higher survival than nonsibling pairing when exposed to one strain of *Metarhizium* (Calleri et al., 2005). The ages of the mature colonies used in this study are unknown; however, the contrasting results observed between incipient and mature colonies suggest that pathogen pressure differentially affects colonies of different ages. Identifying the mechanisms that drive incipient and older colonies to collapse under pathogen threats will require future investigation.

## 5 CONCLUSION

Our results showed no relationship between natural levels of genetic diversity and colony survival against pathogens in *R. flavipes* colonies. This result is consistent with the absence of improved immunity through increased genetic diversity in artificially mixed colonies of this species (Aguero et al., 2020). It should be noted that the results in this study were obtained when colonies were challenged with only two specific pathogen strains within the same genus. Our findings suggest that colony survival, at least toward pathogens from a single genus, may rely more on a specific genetic background, rather than be due to overall genetic diversity of the colony. How genetic diversity affects overall colony survival against the broad range of pathogens that termite colonies naturally face awaits further investigation bridging genes to collective social behavior.

## CONFLICT OF INTEREST

Conflict of interest: None declared.

## AUTHOR CONTRIBUTIONS

**Carlos Aguero:** Data curation (equal); Formal analysis (equal); Investigation (equal); Writing‐original draft (equal). **Pierre‐André Eyer:** Formal analysis (equal); Investigation (lead); Visualization (lead); Writing‐original draft (lead); Writing‐review & editing (lead). **Jason S Martin:** Data curation (equal); Investigation (equal). **Mark S Bulmer:** Conceptualization (lead); Data curation (equal); Investigation (equal); Resources (equal); Writing‐original draft (supporting). **Edward Vargo:** Conceptualization (lead); Resources (supporting); Supervision (lead); Writing‐original draft (supporting); Writing‐review & editing (supporting).

## Supporting information

Supplementary Material

## Data Availability

Data supporting the results of this study have been deposited in Dryad (https://doi.org/10.5061/dryad.fn2z34tsp).
