# Supplementary material for: Natural variation in colony inbreeding does not influence susceptibility to a fungal pathogen in a termite
Source: Ecol Evol. 2021 Mar 10;11(7):3072–83. doi: 10.1002/ece3.7233 (PMC8019025; doi:10.1002/ece3.7233)
Supplement: Supplementary file 1 — Supplementary Material [file ECE3-11-3072-s001.pdf]

Natural variation in colony inbreeding does not influence susceptibility to a fungal pathogen in a termite

Carlos M. Aguero[1], Pierre-André Eyer*[1], Jason S. Martin[2], Mark S. Bulmer[2], and Edward L. Vargo[1]

[1]Department of Entomology, 2143 TAMU, Texas A&M University, College Station, Texas, USA

[2]Department of Biological Sciences, Towson University, 341 Smith Hall, 8000 York Rd., Towson, MD 21252, USA

*Corresponding author:

Pierre-André Eyer

Department of Entomology,

Texas A&M University,

College Station, 77843, Texas, USA

e-mail: pieyer@live.fr

Supplementary Information S1

DNA Extraction:

1. Individual termite workers were placed in a 1.5mL tube.
2. 100 µL of cell lysis solution and 1 µL of proteinase K were added to each tube.
3. Samples were ground in solution using a pestle.
4. Samples were placed in a water bath at 55°C for 3 hours.
5. Samples were removed from the water bath and kept in a freezer at -20°C for 30 minutes.
6. 35 µL of 8M ammonium acetate was then added to thawed samples.
7. Samples were centrifuged for 7 minutes at 10K rpm.
8. The supernatant was pipetted into new tubes containing 100 µL cold isopropanol.
9. Samples were then centrifuged for 5 minutes at 10K rpm.
10. Isopropanol was poured out of tubes, leaving behind pelleted DNA.
11. 400 µL 100% Ethanol was added to each sample.
12. Samples were centrifuged for 5 minutes at 10K rpm.
13. Ethanol was poured out, and samples were placed in a vacuum for 15 minutes to remove any excess alcohol.
14. Dry DNA pellets were resuspended in 100 µL of 1X TE buffer overnight.

PCR Protocols

Multiplex 1

Reagent	volume (µl)/sample
5X Buffer	2.5
RS13 forward	0.42
RS13 reverse	0.42
RS33 forward	0.3
RS33 reverse	0.3
RS62 forward	0.33
RS62 reverse	0.33
Rf15-2 forward	0.22
Rf15-2 reverse	0.22
RS43 forward	0.17
RS43 reverse	0.17
RS16 forward	0.14
RS16 reverse	0.14
Rf6-1 forward	0.06
Rf6-1 reverse	0.06
RS10 forward	0.06
RS10 reverse	0.06
ddH_2O	4.5
Taq	0.1
Extracted DNA	2
Total	12.5

Multiplex 2

Reagent	volume (µl)/sample
5X Buffer	2.1
Rf 24-2 forward	0.25
Rf 24-2 reverse	0.25
ddH_2O	8.8
Taq	0.1
Extracted DNA	1
Total	12.5

Primer sequences and thermocycler programs

<u>Multiplex 1</u>

94°C for 3 min; 7 cycles of 94°C for 30 sec, 62°C for 30 sec (-1°C each cycle), 72°C for 30 sec; then 31 cycles of 94°C for 30 sec, 55°C for 30 sec, 72°C for 30 sec; final extension 72°C for 5 min.

<u>Multiplex 2</u>

94°C for 3 min; 35 cycles of 94°C for 30 sec, 60°C for 60 sec, 72°C for 120 sec; final extension 72°C for 10 min.

Supplementary Information S2

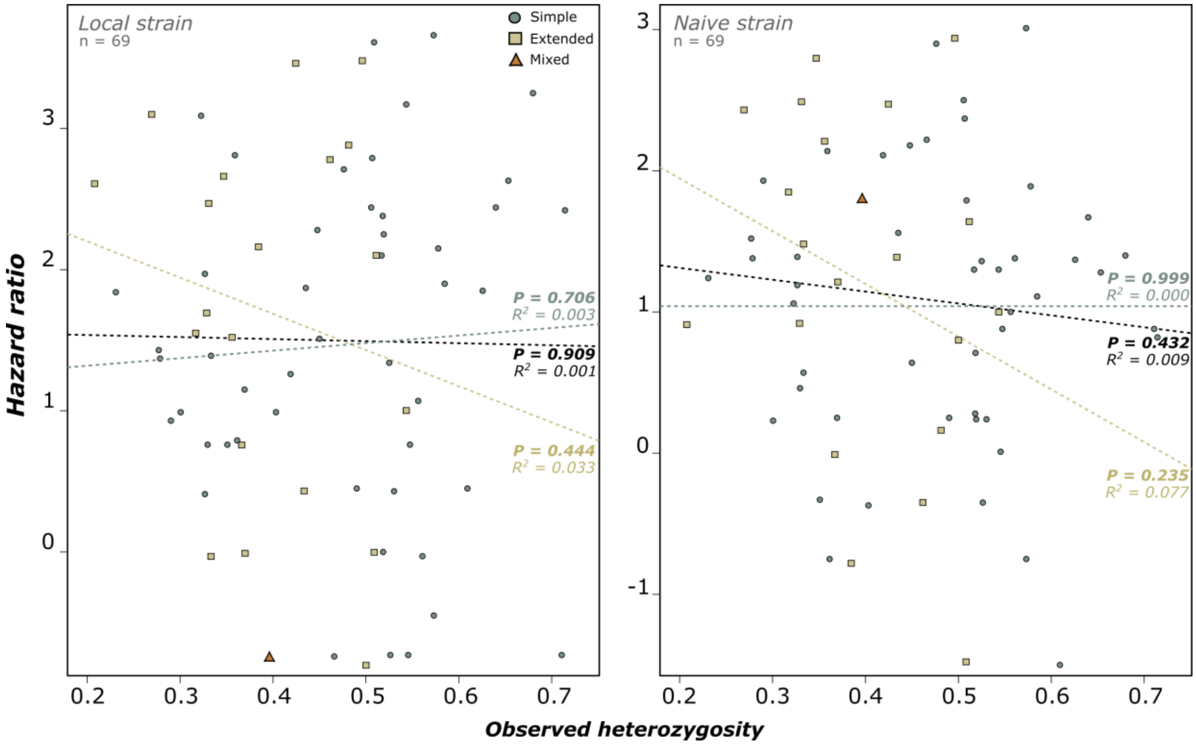

Figure S2: No significant correlation was found between the Observed heterozygosity and the hazard ratio for the local strain (n = 69 colonies, P = 0.909) or the naïve strain (n = 69 colonies, P = 0.432). No correlation were found for the local (P = 0.706 and P = 0.444) and the naïve strain (P = 0.999 and P = 0.235) when the colonies were divided into respectively simple (n = 48 colonies) and extended families (n = 19 colonies).

Supplementary Table T1

Code	Location	Total	Simple	Extended	Mixed
TX1	Lick Creek Park, College Station, TX	7	6	1	0
TX2	Lake Bryan, Bryan, TX	7	4	3	0
NC1	Schenk Forest, Raleigh, NC	9	6	3	0
NC2	Schenk Forest, Raleigh, NC	6	5	1	0
MD1	Gunpowder Falls State Park, Baltimore County, MD	8	5	3	0
MD2	Towson University Field Station, Monkton, MD	10	7	3	0
MA1	Cutler Park, Needham, MA	12	7	5	0
MA2	Middlesex Fells, Medford, MA	10	8	1	1

The location of all sampling sites, with the number of colonies (and their family type) collected from each site.

Supplementary Table T2

Generalized linear models

<u>Hazard ratio for the local strain ~ (FIC + Family type)</u>

	Estimate	*P*-value	
(Intercept)	1.8089	1.23E-07	***
FIC	1.5878	0.3777	
FamilyMixed	-2.4013	0.0596	.
FamilySimple	-0.7015	0.2424	
FIC:FamilyMixed	NA	NA	
FIC:FamilySimple	-2.6938	0.2491	

<u>Hazard ratio for the local strain ~ (FIC + Family type)</u>

	Estimate	*P*-value	
(Intercept)	1.3991	1.13E-06	***
FIC	2.6157	0.0921	.
FamilyMixed	0.6541	0.5438	
FamilySimple	-0.3889	0.4475	
FIC:FamilyMixed	NA	NA	
FIC:FamilySimple	-2.7098	0.1764	

Results from generalized linear model was used to determine whether all of the inbreeding coefficient (F_{IC}) and family type individually influenced the hazard ratios, or if there were any interaction effects between these two factors.
